# Supporting Australian clinical learners in a collaborative clusters education model: a mixed methods study

**DOI:** 10.1186/s12912-020-00451-9

**Published:** 2020-06-26

**Authors:** Thea F. van de Mortel, Lyn Armit, Brenton Shanahan, Judith Needham, Candy Brown, Eileen Grafton, Michelle Havell, Amanda Henderson, Laurie Grealish

**Affiliations:** 1 Professor & Deputy Head of School (Learning and Teaching), School of Nursing and Midwifery, Griffith University, Parklands Drive, Southport, QLD 4222 Australia; 2 Director of Nursing, Nursing/Midwifery Education and Research Unit, Gold Coast University Hospital, Southport, Australia; 3 Adjunct Professor School of Nursing and Midwifery, Griffith University, Southport, QLD 4222 Australia; 4 ADON Education Programs, Nursing/Midwifery Education and Research Unit, Gold Coast Health, Gold Coast, QLD Australia; 5 Director, Clinical Practice Office, School of Nursing and Midwifery, Griffith University, Meadowbrook, QLD Australia; 6 Nurse Educator, Nursing/Midwifery Education and Research Unit, Gold Coast University Hospital, Gold Coast, QLD Australia; 7 School of Nursing and Midwifery, Griffith University, Meadowbrook, QLD Australia; 8 Nurse Unit Manager, C2E Digestive Health, Surgical, Anaesthetics, and Procedural Services, Gold Coast University Hospital, Gold Coast, Australia; 9 Professor, School of Nursing and Midwifery, Griffith University, Nathan, Australia; 10 Nursing Director, Nursing Practice Development Unit, Princess Alexandra Hospital, Brisbane, QLD Australia; 11 Associate Professor, School of Nursing and Midwifery, Griffith University & Gold Coast Hospital and Health Service, Southport, QLD Australia; 12 Menzies Health Institute Queensland, Griffith University, Gold Coast, QLD Australia

**Keywords:** Education, clinical, Students, nursing, Clinical supervision, Registered nurses

## Abstract

**Background:**

Nursing student numbers have risen in response to projected registered nurse shortfalls, increasing numbers of new graduates requiring transitional support and pressure on clinical placements. A Collaborative Clusters Education Model, in which Entry to Practice facilitators coach ward-based registered nurses to support students’ and new graduates’ learning, may address placement capacity. The research aim was to evaluate the acceptability of the Collaborative Clusters Education Model to stakeholders by examining their perceptions of the facilitators and barriers to the model in its implementation.

**Methods:**

A convergent mixed methods evaluation approach was adopted. The study took place in a large Australian health service in south-east Queensland. Participants included Bachelor of Nursing students, Entry to Practice facilitators, ward-based registered nurses, academics and new graduates. A mixed methods design was used. Elements included an online survey of nursing students, and interviews with new graduates, Entry to Practice facilitators, ward-based registered nurses, and academics. Descriptive statistics were calculated on quantitative data. Thematic analysis was conducted on qualitative data.

**Results:**

Participants included 134 (of 990) nursing students (response rate 13.5%), five new graduates, seven Entry to Practice facilitators, four registered nurses, and three nurse academics. Students rated facilitators’ effectiveness highly (4.43/5 ± 0.75), although this finding is tempered by a low response rate (13.5%). For learners, the model provided access to learning experiences, although preferences for sources of support differed between students and new graduates, and further clarification of responsibilities was required. For other stakeholders, three themes emerged: students’ and new graduates’ integration into the workplace can promote learning; tensions arise in new ways to approach performance assessment; and aligning expectations requires high levels of communication.

**Conclusions:**

This evaluation found that acceptability was good but at risk from limited clarity around roles and responsibilities. Further research into this model is recommended.

## Background

There are various drivers to promote innovation in clinical education for nursing students. In one jurisdiction, the international health workforce shortage projections [1] were a trigger for innovation. University leaders have increased intakes into baccalaureate nursing programs to meet workforce demand [1]. These higher student enrolments are also associated with increased numbers of new graduates, who require transition support upon entering the workforce [2].

However, a key challenge to the supply of undergraduate nursing students is difficulty obtaining sufficient clinical placements [3]. To further complicate this situation, health agencies are experiencing higher patient acuity and shorter lengths of stay [4], which potentially puts pressure on staff and supervision capacity. These factors challenge higher education providers and health services to co-develop clinical education models that increase placement capacity without adversely affecting learning quality and care of health service consumers.

Bachelor of Nursing (BN) curricula are also increasingly based upon constructivist approaches to learning that assume that learners process new knowledge by relating it to that acquired in previous experiences, and make meaning of new information through social interactions [5, 6]. While nursing students in bachelor’s degrees have been supernumerary on clinical placement – ie., extra to standard ward staffing and not allocated a patient load [7] - models that embed students as part of the team develop the workplace as a community of practice, and provide opportunities to make meaning in social collaboration with others, thus improving learning [8].

Increasing the social capital of clinical contexts by ‘creating connections, enhancing relationships and fostering knowledge sharing’ has demonstrated success in increasing placement numbers and student learning opportunities during clinical placement [9]. In one jurisdiction, baccalaureate students were refigured from supernumerary to members of the nursing team, distributed across hospital wards and community-based services. Clinical learning facilitators, called Entry to Practice (E2P) facilitators, supported the learning requirements for students and new graduates across the health service. This study describes the evaluation of the acceptability of this innovative approach, called the Collaborative Clusters Education Model (CCEM).

Various clinical placement models are utilised in nursing education [10]. For example, some schools employ clinical facilitators (usually external to the host unit) who work with students, usually on a 1:8 ratio, to assist and assess their learning. The facilitator is supernumerary - additional to the standard nursing workforce for the unit/ward. In other models, students are partnered directly with Registered Nurses (RNs) who are providing patient care over a number of shifts. Variations are tailored to suit specific industry provisions [10]. Facilitator model limitations include a potential lack of clinical expertise in a specialty area and staffing and cost restrictions associated with the standard 1:8 facilitator to student ratio, and limitations of models where ward-based RNs support student learning include RNs’ lack of educational expertise, and situations in which work overtakes learning [11].

Another educational model, the Dedicated Education Unit (DEU) is emerging [10]. The DEU - established in Australia and now used internationally [12] - overcomes the key limitations of these models by incorporating the best elements of both [12]. In the DEU, a clinical facilitator works with ward-based RNs to develop their ability to facilitate learning while removing the restrictive staff student ratio [13]. The ward-based RNs are clinical experts while facilitators understand educational best practice to guide ward-based RNs help students learn. Principles of the DEU align with best clinical learning practice through encouraging the entire clinical nursing team to engage students in learning, while assigning specific academic responsibilities, such as assessment, to a designated person from the education facility [14]. However, the DEU was developed for integrated placements in which students attend placement several days per week over the teaching semester, while the majority of Australian nursing students complete block placements, e.g., full time placements for shorter set periods.

Until 2015, our health service employed traditional clinical education models that utilised a 1:8 ratio. Clinical facilitators were health service staff, employed in the facilitator role. Training for the clinical facilitators was shared between the university and health service.

In 2013 and 2014, an integrated practice model, where 60 year-three nursing students completed a roster of continuous shifts linked to nominated ward-based RNs was trialled. Clinical facilitators supported the RNs and students without a 1:8 ratio of facilitators to students. This trial was the impetus for changing the focus of student placement support to a new model, more like the DEU.

### The collaborative clusters education model

**Fig. 1:**
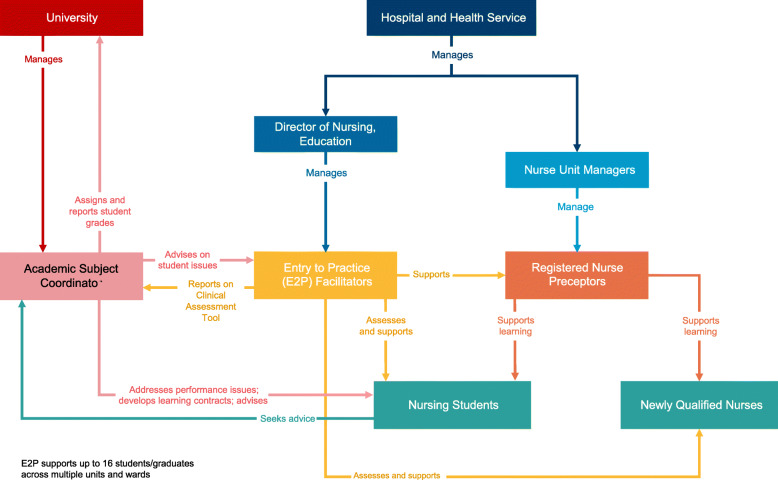
Collaborative clusters education model

The CCEM, was adapted from the DEU to incorporate a block placement design. In the CCEM, the facilitator continues to be a health service employee, which is a key difference from the DEU, where the facilitator is usually a university employee. The facilitator is assigned to several clinical wards/units and oversees up to 16 students and/or new graduates across those wards concurrently, thus the use of the term ‘cluster’ in relation to the model (Fig. 1).

More than one clinical facilitator may share responsibility for students’ learning depending on shifts. The clinical facilitators work closely with ward-based RNs. In terms of student performance, the clinical facilitators liaise with academic subject convenors and RNs, as well as with each other to monitor each student’s experience. Hence the use of the term ‘collaborative’ in relation to the model.

The clinical facilitator supports RNs to engage with students and new graduates assimilating into the clinical setting, providing guidance to RNs on appropriate learning activities. Consistent with earlier models, the need for RNs to support others’ learning is an important professional responsibility [15] in the CCEM. In addition to guiding RNs, the clinical facilitator conducts formative and summative assessment of learners, and provides feedback on their performance, and documents learner achievements. The clinical facilitator’s expertise is grounded in work-based learning rather than specialist clinical expertise.

In the CCEM, the responsibility of the academic staff from the university is to: review and approve the clinical facilitator’s assessment of students’ performance using a standard Clinical Assessment Tool; assign a course grade; and manage any student performance issues. For example, students who are not meeting performance standards are provided with mid placement feedback and a ‘formative review’ in which their performance is discussed with the academic subject convenor, and a learning contract is developed to guide future performance. For more information on the model, see [16]. To make the CCEM facilitators easily identifiable to students, new graduates and staff, they wear a different uniform and are called Entry to Practice (E2P) facilitators. The CCEM was implemented in the health service in 2015 over a three-month period.

The transition to the CCEM required shifting students’ and RNs’ expectations about access to learning support, which is acknowledged as challenging [17]. A process involving key stakeholders was implemented to bring about this change. A reference group, consisting of researchers, clinicians, educators and students, provided governance of the project, including identification of implementation activities. Preparations included briefings for Nurse Unit Managers and masterclasses for the E2P facilitators on how to work within the model. Earlier work established that the CCEM was feasible [15]. Understanding stakeholder acceptability of the model to support undergraduate nursing student learning is critical to sustainability [18].

## Methods

### Design

This study used a convergent parallel mixed methods design that included a survey and a descriptive qualitative approach [19]. The convergent design allows the collection of different, but complementary data on the same topic to be analysed during the same phase of the research process, and then merges the results into an overall interpretation [19]. The topic for this research was the acceptability of the CCEM for stakeholders, through examining their experiences of the facilitation of their learning within the model and their perceptions of factors that enhanced or created barriers to successful implementation of the model.

### Participants and recruitment

**Fig. 2:**
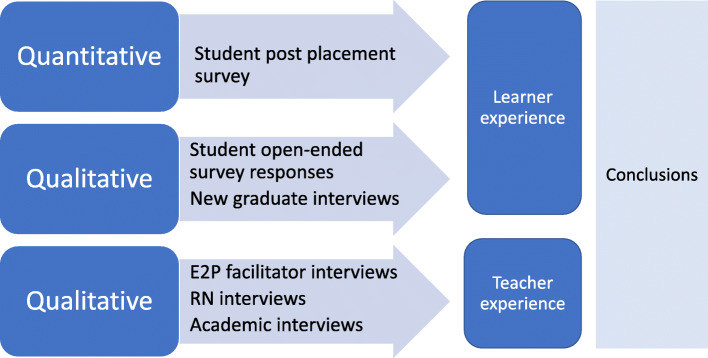
Data collection in parallel mixed methods design

The study was set in a tertiary level Australian health service in 2015 following initial implementation of the model. Participants were aged 17–70 years and included identified stakeholders: undergraduate Bachelor of Nursing students, new graduates, ward-based RNs, E2P facilitators, and academics who convened the students’ clinical courses whilst on placement. The data sources for the study are outlined in Fig. 2.

Students were invited by email to complete an anonymous online questionnaire. New graduates and ward-based RNs were invited by flyer to participate in an interview. The E2P facilitators were provided with information at a scheduled workshop. Nurse academics were invited to participate in interviews via email. All were provided with an information sheet and consent form.

### Data collection

The student questionnaire was the standard post-placement survey distributed to students who had completed a placement by the University’s Professional Practice Office within 2 weeks of placement completion (supplementary file). It had 19 items on a 5-point Likert scale (1 = strongly disagree; 5 = strongly agree) focused on students’ perceptions of the qualities and behaviours of the E2P facilitators (14 items) and ward-based RNs (5 items) supporting them. Within the survey, students were also invited to respond to three open-ended questions about their experience including what the strengths of their facilitators were, what were areas for improvement and other comments they had about the facilitation of the placement.

New graduates, RNs, E2P facilitators, and academics were interviewed individually for approximately 30 min and invited to describe how they participated in the CCEM, their experiences of learning, or supporting learning, and their perceptions of factors that enhanced, or were barriers to, CCEM implementation. Interviews were conducted by a research assistant or team member (LG), and held by telephone or in the participant’s workplace as per the participant’s preference. The interviews were digitally audio-recorded and professionally transcribed.

### Data analysis

For the student survey, descriptive statistics were calculated for fixed response questions using IBM SPSS Statistics version 23. Students with missing data were excluded from the analyses. A comparison of means for second and third year students using an independent samples t-test demonstrated no significant differences (*p* = 0.142–0.682) between year level groups, so the data were pooled for the final analysis. Scale reliability was determined using Cronbach’s alpha.

Thematic analysis was used for the students’ open-ended survey comments and all of the interview data. This involved becoming familiar with the data through re-reading transcripts, using a systematic process to code data, clustering codes into named themes, reviewing these, and reporting themes along with salient quotes [20]. The student comments and new graduate interviews were coded and thematically analysed separately and then the findings were combined to arrive at overall themes from the learner perspective.

The interview data from the RNs, E2P facilitators and academic nurses were analysed as a group with shared experience of supporting student learning in the workplace. Thematic analysis, as described in the previous section, was used. Trustworthiness of the findings was confirmed through the selection of participants with experience in the CCEM, rich variation in perspectives provided by a relatively diverse sample of participants and use of data saturation for the interview data [21]. Data saturation occurred when no new themes appeared. Themes were derived by two researchers independently, which were then discussed to determine a final list. A stakeholder reference group reviewed processes and findings to encourage researcher reflexivity.

The results of the data produced by each stakeholder group was then merged to arrive at an understanding of stakeholders’ acceptability of the model. To do this, data was initially presented to the stakeholder reference group, who discussed the data, and together with researchers, identified areas of convergence, divergence, and relatedness in order to produce a more complete understanding [19].

### Ethics approval

Ethics approval was obtained from the health service (15/QGC/152) and university (NRS/30/15/HREC) Human Research Ethics Committees. Interview participants provided informed written consent. Confidentiality was assured through de-identifying transcripts. Study data are held in secure electronic files. Completion of the anonymous online survey was considered consent.

## Results

The survey was completed by 134 of 990 nursing students placed in the health service during the study period (response rate 13.5%): 85 were in the second year and 49 in the third year of their three-year Bachelor of Nursing program. The majority were aged 18–25 (56.7%). One hundred and thirteen Bachelor of Nursing students provided responses to open ended questions on placement facilitation. Five new graduates, seven E2P facilitators, four ward-based RNs, and three nurse academics participated in interviews.

### Student and new graduate responses

Scale reliability ranged from 0.77–0.90. Students rated the effectiveness of their E2P facilitators highly (4.43/5 ± 0.75) and were highly satisfied with the behaviour and qualities of the ward-based RNs (Table 1) and E2P facilitators (Tables 1 and 2).

**Table 1:** Students’ perceptions of Registered Nurse (RN) and Entry to Practice Facilitator (E2P) qualities on a Likert scale (1 = strongly disagree; 5 = strongly agree)

Item	RN Mean (± s.d.) (***n*** = 133)	E2P Facilitators Mean (± s.d.) (***n*** = 128)
Professionalism	4.4 (± 0.8)	4.6 (± 0.7)
Supported my learning experience	4.5 (± 0.7)	4.5 (± 0.9)
Willingness to provide feedback	4.4 (± 0.8)	4.6 (± 0.6)
Well qualified with up-to-date knowledge and practice	4.5 (± 0.7)	4.7 (± 0.6)
Flexibility in facilitating my learning experience	4.5 (± 0.7)	4.4 (± 0.9)
Effective management of group dynamics	N/A	4.5 (± 0.8)
Effective utilisation of de-briefing sessions	N/A	4.4 (± 1.0)
Total	4.5 (± 0.7)	4.5 (± 0.7)

**Table 2:** Students’ perceptions of the Entry to Practice Facilitator’s behaviours on a Likert scale (1 = strongly disagree; 5 = strongly agree)

Item	Mean (± s.d.) (***n*** = 128)
Successfully orientated me to the health facility/service, staff and emergency procedures	4.4 (± 0.9)
Encouraged active learning using critical thinking	4.5 (± 0.7)
Helped me achieve my learning objectives.	4.4 (± 0.8)
Promoted the integration of on and off campus learning in relation to key learning concepts and new learning material.	4.4 (± 0.8)
Assisted me to create and develop effective communication strategies in the clinical environment.	4.4 (± 0.8)
Encouraged me to contribute to the clinical assessment process.	4.5 (± 0.9)
Scale average	4.4 (± 0.7)

Students’ open-ended survey comments and new graduate interview findings resonated with each other, with both data sets grouped into three areas: ‘access to learning’; ‘source of support’, and ‘lack of clarity about facilitation.’

### Access to learning

Both students (SN) and new graduates (NG) indicated that the model provided good learning opportunities, with access to authentic experiences. For example:

‘I had a great experience with all staff and facilitators and…have learned a lot while on placement’ (SN86)

‘[it provided a] chance to work with a number of different nurses…that was very good because you got to see different styles of nursing’ (NG7)

### Source of support

Views on the level of learning support differed across the two groups. For example, students were more likely to report the absence of E2P facilitators in comments such as:

‘more support from the [E2P] facilitators would be good. I think they are too busy with too many students and some more practical hands on with a facilitator would be better’ (SN 86).

New graduates largely felt well supported by the E2P facilitators, and emphasised the support from ward nurses:

‘The [E2P] facilitators…give you your own chance to learn and then there’s support if you need it, and if they think that you’re obviously not doing as well as what you could be, they’re there to tell you that and talk you through things and give you feedback and advice to fix anything so I think there would just be a perfect amount of support’ (NG4)

### Lack of clarity about facilitation

Students appeared to be confounded by the E2P teams and to seek one person to be ‘their facilitator’ as per previous models. This was manifest in comments such as:

‘it was a draining experience not knowing who was the actual [E2P] facilitator’ (SN34).

In summary, the learning was positive for both students and new graduates, but students appeared to be more focused on support from E2P facilitators and new graduates more focused on support from the ward-based RNs. The transition to multiple E2P facilitators was confusing for students.

### Academics, RNs and E2P facilitators

Thematic analysis of the interviews with ward-based RNs, E2P facilitators, and academics revealed three main themes: 1) students’ and new graduates’ integration into the workplace can promote learning; 2) tensions arise in new ways to approach performance assessment; and 3) aligning expectations requires high levels of communication.

#### Students’ and new graduates’ integration into the workplace can promote learning

Ward-based RNs valued the multiple perspectives that students would learn by working with different staff and indicated that the CCEM potentially reduced the impacts of personality clashes that arose in traditional supervisory relationships. Although some expressed concern about the impacts of a lack of continuous facilitator supervision:

‘they get a variety [of E2Ps]…so if there’s a personality clash with a facilitator and a student there isn’t that issue because you've got quite a few that cover the wards’ (RN1)

‘With the old model…if things were quite busy on the ward the facilitator would come up and spend a bit more time with the students to work on any needs…I worry that the students aren’t getting the support in that sense because the facilitator does have so many students to cover…[some may] ‘slip through the cracks’ (RN1)

E2P facilitators and academic nurses agreed that the model encouraged greater integration of learners into the ward culture, developing the student-RN relationship and students’ problem-solving and independence:

‘With the new model [learners] integrate into the ward much, much better. They become part of the team because you’re not on the floor all the time with nurses [saying] ‘your facilitator can do that with you’…now the unit staff are taking ownership…I think it’s a better experience for [learners] in that they…actually become part of that unit…they have to develop their problem-solving skills in that situation a lot better than the old model where you are problem-solving for them a lot’ (E2P7)

‘it definitely fosters the students to be independent, and to seek their own learning opportunities’ (E2P5)

[Third years] ‘felt that they were becoming part of the team and that they could troubleshoot and communicate with other staff and felt comfortable about that … (Academic Nurse (AN) 1)

However, this was also identified as an area of concern for those students who may not be strong communicators or who are earlier in their career trajectory, such as second year students:

‘… felt that those who weren’t as confident … would have difficulty in asking for help from anyone else but their facilitator, so they were thinking it would be more difficult for the second years’ (AN1).

#### Tensions arise in new ways to approach assessment of performance

E2P facilitators identified other benefits with the CCEM, including better cover for sick leave, a better relationship with ward-based RNs, and broadening their perspectives of student performance through sharing feedback during handover:

‘you’ve got a lot of people working with the same students, so you’ve got different perspectives of how that…group of students are performing’ (E2P2)

There were continuities in how student learning was facilitated such as ‘giving feedback…[investigating] before you give that feedback [to moderate it]…timekeeper for sick leave…written assessments…troubleshooting issues…role model and mentor’, and encouraging student reflection (E2P5); ‘helping the buddies [RNs], being able to educate and giving them support’ (E2P2), ‘building those relationships with…all the staff’, ‘identifying issues quickly (E2P6); and ‘[supporting] the students when they need it clinically and emotionally’ (E2P3).

However, there were also tensions, particularly in relation to the assessment of student performance such as: trying to find students and the RNs supporting them (E2P1, E2P6), feeling rushed (E2P1, E2P4), and insufficient time to gather enough information to inform assessment (E2P1, E2P5, E2P 6, E2P7). Academics were also concerned about assessment:

‘the [Clinical Assessment Tools] that came back…[had] generic comments…I really want to make sure that [students are] clinically sound and I can’t do that when the comments are just not there’ (AN3).

In summary, while learning appeared to be enhanced by the CCEM, the tensions associated with multiple staff and perspectives, particularly around processes for providing feedback on performance (assessment), required further development.

#### Aligning expectations requires high levels of communication

Good communication and role delineation were considered integral to the success of the model, for example:

‘constantly keeping that communication line open with those key people in that area, [which is] pivotal...in making that student’s clinical placement a positive experience just by negotiating and ensuring that they’re learning what they need to learn’ (E2P7)

‘It’s important that the [ward-based RNs] understand their education role more effectively…[they think] if the students [or new grads] are getting on with it and look like they are doing it, they must be great. But there’s not that in depth asking of questions or delving a little bit more to see whether or not that is the case’ (E2P1)

‘Maybe we need to have a bit more discussion on what is expected [regarding] whether you see every student every shift’ (E2P1)

Processes for communication across the stakeholder groups were negotiated through the reference group activities and discussions. However, further communication appeared to be required. For example, academic nurses noted that third year students were expecting the supervisory model they had previously experienced, and this was potentially related to insufficient communication about the changes:

‘I [had] complaints from students because they didn’t understand why they couldn’t actually get their [E2P] facilitators as often and now I understand that they should not be looking to their facilitators as much for the education as their [ward-based RN], but that wasn’t the understanding then when that class was on [placement]’ (AN3)

Academics continued to monitor students on placement. The academics reported feeling frustration as they adjusted to the model. For example, having more than one E2P facilitator confounded communication:

‘it’s very frustrating from my end trying to contact the people who are looking after my students’ (AN3)

As the model was transitioning, E2P facilitators adjusted as well, stressing their perception that their role is now educational support and liaison rather than clinical expert:

‘I think someone from general could go to mental health and step into the role if they had to in this model. It’s the nurses who are the specialists in that area…so they do the teaching, whereas this role now is more liaising and anyone can do that if they’ve got the skills that they need for that, so I think the role is better’ (E2P3)

In summary, the transition to the CCEM required alignment of stakeholder expectations and the communication strategies to achieve alignment were inadequate to support the transition.

The students who responded to the survey strongly agreed that the E2P facilitators supported their learning and scored E2Ps and ward-based RNs highly on professionalism, currency of knowledge, flexibility, and willingness to provide feedback. For new graduates, the opportunity to work with multiple nurses with intermittent E2P facilitator support provided structured support and an opportunity to learn about different styles of nursing. However, students appeared to prefer, and expected, supervision from one E2P facilitator whereas new graduates preferred learning from the staff. The acceptability of the CCEM for nursing students appeared limited by their preference for a closer relationship with a single E2P facilitator.

Academics, E2P facilitators and RNs appeared to find the model acceptable, noting that improved communication of expectations is required. They valued the increased independence of more senior students and new graduates in their learning, noting that more junior students may require additional support. Although there was general agreement that learner exposure to multiple perspectives is a strength of the model, processes to communicate about individual learner performance were confounded by multiple stakeholders and required the establishment of clear lines of communication in order to sustain the model.

## Discussion

This research focused on the acceptability of the CCEM during implementation to determine what may still be required for sustainability of the CCEM program.

The students who responded to the survey valued the roles of the E2P facilitator and RNs, highly ranking professionalism, knowledge, flexibility and feedback. These highly ranked behaviours are recognised as important to engage students in workplace learning [22]. The CCEM appears to be acceptable in terms of access to meaningful learning experiences.

New graduates found the model acceptable, with the opportunity to work with a variety of staff valued. This is consistent with nurse transition research that suggests they are working to find their professional selves, distinguish themselves from others and be accepted by the professional culture in the first 4 months [23].

The integration of students and new graduates into the ward culture occurred relatively easily during the transition to the CCEM. This experience is similar to the introduction of the DEU model as increased exposure provides greater learning opportunities [24]. New graduates preferred learning from the staff, however, students expressed a preference for continuous support from a single facilitator. Further work is required to determine whether this preference is related to poor communication about the changed model, concerns about assessment, specifically who will assess the student’s performance, or a genuine preference for a single person to provide consistent feedback.

Assessment of student performance emerged as a critical area for further development. The lack of a consistent E2P or ward-based RN was a source of concern for students and academic nurses. Clear roles, responsibilities and patterns of communication are required in order to achieve sustained acceptability of the CCEM. An unexpected benefit in the assessment process was the development of E2Ps as assessors through sharing their perceptions of student performance within their teams. The value of this form of moderation and skill development in assessment of students’ clinical performance requires further investigation.

Acceptability of the model was not achieved for academic nurses, who were disappointed with the generic information provided on student clinical performance assessment instruments. To ensure high quality nursing graduates, further research into how to enhance clinical assessment, and in particular providing specific information about areas for development is required. Practical developments may include processes for gathering data on student performance from ward-based RNs, collating data in a way that can be accessed by multiple E2P facilitators, or simply be a lack of clarity about accountability for specific student assessments between facilitators. Determining the usefulness of standardised instruments, with relatively strong reliability and validity, such as the Australian Nursing Standards Assessment Tool [25], in this model is required.

Lack of alignment between stakeholder expectations and what the model provided was a key challenge to acceptability. Students were briefly introduced to the model in their orientation but were expecting to work more closely with a single facilitator as they had done previously. The lack of E2P facilitator continuity is a risk to acceptability as poor continuity is a potential stressor [26] and must be addressed if the CCEM is to be sustained.

While there were new opportunities for communication between E2P facilitators and ward-based RNs, communication between stakeholders was often inadequate, potentially reducing acceptability. Communication can be improved through an established communication plan including:

1. a poster with the photos and names of E2P facilitators mounted in wards;
2. a shared folder so that data collected from direct observation and RN reports can be recorded and reviewed by facilitator teams;
3. regular meetings between E2P teams to discuss student progress, and plan and evaluate development activities for ward-based RNs supporting students.

In regard to stakeholder expectations, the E2P facilitators were clear about their role but this clarity was not shared by other stakeholders. In particular, the role of the ward-based RN was unclear. To improve acceptability, it will be important to build on the foundational relationship between E2P facilitators and ward-based RNs. E2P facilitators can work with nursing teams to identify appropriate nursing activities in their specific health service settings. Identifying activities for students at different stages can support scaffolded learning, a key aim for clinical placement [27]. Other strategies are to encourage RNs supporting students to attend regular workshops on giving and receiving feedback, work-based pedagogies, and clinical leadership. Workshops or masterclasses for E2P facilitators can also focus on skills such as coach for RNs to support student learning as part of their usual work practices [28].

Of note, the RNs preferred the E2P facilitator to supervise student practice, particularly when the workload was high. Further training for ward staff on how to incorporate students into their teams and delegate appropriate nursing responsibilities to enhance learning, is required to augment acceptability.

### Limitations

There were limitations to this study. Merging results of quantitative and qualitative data in a meaningful way can be challenging. This study was purposefully designed with a focus on the same concept, learners in the workplace, which is acknowledged as important in convergent designs [19]. Using experienced researchers is critical in this design [19]. Data analysis was supervised by doctorally prepared and experienced researchers (TM quantitative; LG qualitative).

While survey responses were anonymous, research indicates that responses to both surveys and interviews may be subject to social desirability response bias [29], which may skew results. Historically, the student response to the clinical experience survey is usually around 15–25%, thus the low response rate was not surprising. To manage our expectation of a low response, we also considered the open-ended comments and included interviews with new graduates.

While the sample for the qualitative component was small (*n* = 19), Braun and Clarke [30], suggest that 5–15 participants is adequate for a small qualitative study, and qualitative studies do not aim for generalisability. Using multiple data sources, including multiple stakeholders in the CCEM provided a rich source of data that may address these limitations to some extent, although further research with students and new graduate nurses in this model is required to assure acceptability in the future and to determine whether these results could be meaningful for other health services.

## Conclusions

The CCEM was designed to be like a DEU, where students learn by engagement in authentic clinical practice. While the E2P facilitators described their role as supporting ward-based RNs to increase their skills in facilitating student learning from practice, continued development of their role in developing their allocated wards as learning units is needed, including the identification of suitable cases and learning strategies, and mastery of the process of continuous feedback on performance to ensure sustained acceptability. The CCEM provides an opportunity to gain the benefits for student learning that are found in a DEU while providing for block placements, such as student engagement in authentic work as part of a team, and development of work-based pedagogies. Given a relatively short implementation timeframe, initial evaluation focused on acceptability as this was critical to sustainability. The rapid transition did contribute to communication deficits that require continued monitoring for improvement. For others interested in this model, we recommend a good communication plan, and attending to formal structures and processes between stakeholder groups. Future evaluations will include the appropriateness of the model for specific areas, what elements enhance adoption, fidelity of the model in different settings, as well as cost and sustainability.

## Supplementary information

**Additional file 1.** Placement survey. Copy of the placement survey.

## Data Availability

The dataset contains some identifying information of wards and clinical facilitators and therefore will not be shared. The survey questions can be found in the supplementary file named Additional file 1.

## Abbreviations

AN: Academic Nurse
BN: Bachelor of Nursing
CCEM: Collaborative Clusters Education Model
DEU: Dedicated Education Unit
E2P: Entry to Practice
NG: New Graduate
RNs: Registered Nurses
SN: Student Nurse
