# Supplementary material for: Supporting Australian clinical learners in a collaborative clusters education model: a mixed methods study
Source: BMC Nurs. 2020 Jun 26;19:57. doi: 10.1186/s12912-020-00451-9 (PMC7318499; doi:10.1186/s12912-020-00451-9)
Supplement: Supplementary file 1 — Additional file 1. Placement survey. Copy of the placement survey. [file 12912_2020_451_MOESM1_ESM.docx]

**Placement survey**

*The following questions pertain specifically to the ward staff. Feedback about your Clinical Facilitator will be covered later.*

**1. Please identify the name of the hospital or health service/ward where you did your clinical placement.**

|  |
| --- |

**2. Indicate your level of agreement with the following statement.**

(strongly disagree, disagree, unsure, agree, strongly agree)

The ward was appropriate for the set clinical objectives.

**3. The ward registered nurses demonstrated the following behaviours/qualities:**

(strongly disagree, disagree, unsure, agree, strongly agree)

Willingness to provide feedback

Professionalism

Flexibility in facilitating my learning experience

High qualification with up to date knowledge and practice

Support of my learning experience

*The last few questions pertain to your experience with your CLINICAL FACILITATOR. Please reflect on the behaviours and characteristics demonstrated by this person during your placement.*

**4. Please name your Clinical Facilitator.**

**5. Please select your level of agreement with the following statements about your Clinical Facilitator's personal qualities.**

(strongly disagree, disagree, unsure, agree, strongly agree)

**My Clinical Facilitator demonstrated...**

Support of my personal learning experience

High qualifications with up to date knowledge and practice

Effective utilization of debriefing sessions

Professionalism

Flexibility in facilitating my learning experience

Willingness to provide feedback

Effective management of group dynamics

**6. Please select your level of agreement with the following statements about your Clinical Facilitator's specific actions or behaviours**

**My Clinical Facilitator...**

Encouraged active learning using critical thinking

Encouraged me to contribute to the clinical assessment process

Successfully orientated me to the health facility/service and staff

Promoted the integration of on and off campus learning in relation to key learning concepts and new learning material.

Helped me achieve my learning objectives

Assisted me to create and develop effective communication strategies in the clinical environment

**7. All things considered, how would you rate your Clinical Facilitator's overall effectiveness?**

| Unacceptable  Poor  Satisfactory  Good  Outstanding |
| --- |

**8. What were your Clinical Facilitator's most prominent strengths?**

**9. What areas of improvement can you suggest for your Clinical Facilitator?**

**10. Please enter any other comments about your experience with your Clinical Facilitator here.**

**11. Please select your age group below.**

| 18 – 25  26 – 35  35 - 45  46+ |
| --- |
